# Microbial production of lipid-protein vesicles using enveloped bacteriophage phi6

**DOI:** 10.1186/s12934-019-1079-z

**Published:** 2019-02-07

**Authors:** Outi L. Lyytinen, Daria Starkova, Minna M. Poranen

**Affiliations:** 1 Molecular and Integrative Biosciences Research Programme, Faculty of Biological and Environmental Sciences, University of Helsinki, 00014 Helsinki, Finland; 2 Present Address: Laboratory of Molecular Epidemiology and Evolutionary Genetics, St. Petersburg Pasteur Institute, Mira St. 14, St. Petersburg, 197101 Russia

**Keywords:** Vesicle production, *Escherichia coli*, Pseudomonas phage phi6, Viral envelope formation, Major envelope protein P9, Non-structural protein P12

## Abstract

**Background:**

Cystoviruses have a phospholipid envelope around their nucleocapsid. Such a feature is unique among bacterial viruses (i.e., bacteriophages) and the mechanisms of virion envelopment within a bacterial host are largely unknown. The cystovirus Pseudomonas phage phi6 has an envelope that harbors five viral membrane proteins and phospholipids derived from the cytoplasmic membrane of its Gram-negative host. The phi6 major envelope protein P9 and the non-structural protein P12 are essential for the envelopment of its virions. Co-expression of P9 and P12 in a *Pseudomonas* host results in the formation of intracellular vesicles that are potential intermediates in the phi6 virion assembly pathway. This study evaluated the minimum requirements for the formation of phi6-specific vesicles and the possibility to localize P9-tagged heterologous proteins into such structures in *Escherichia coli*.

**Results:**

Using transmission electron microscopy, we detected membranous structures in the cytoplasm of *E. coli* cells expressing P9. The density of the P9-specific membrane fraction was lower (approximately 1.13 g/cm^3^ in sucrose) than the densities of the bacterial cytoplasmic and outer membrane fractions. A P9-GFP fusion protein was used to study the targeting of heterologous proteins into P9 vesicles. Production of the GFP-tagged P9 vesicles required P12, which protected the fusion protein against proteolytic cleavage. Isolated vesicles contained predominantly P9-GFP, suggesting selective incorporation of P9-tagged fusion proteins into the vesicles.

**Conclusions:**

Our results demonstrate that the phi6 major envelope protein P9 can trigger formation of cytoplasmic membrane structures in *E. coli* in the absence of any other viral protein. Intracellular membrane structures are rare in bacteria, thus making them ideal chasses for cell-based vesicle production. The possibility to locate heterologous proteins into the P9-lipid vesicles facilitates the production of vesicular structures with novel properties. Such products have potential use in biotechnology and biomedicine.

**Electronic supplementary material:**

The online version of this article (10.1186/s12934-019-1079-z) contains supplementary material, which is available to authorized users.

## Background

Vesicles are spherical membrane structures composed of a lipid bilayer enclosing aqueous material. The bilayer is composed of phospholipids and is typically embedded with membrane proteins. The inner fluid can contain cargo molecules such as nucleic acids or soluble proteins. Eukaryotic cells have elaborate cell signaling and trafficking systems based on different kinds of vesicle structures and membrane organelles. In contrast, vesicles and other inner membrane structures are rare in bacteria. Examples of cytoplasmic membrane organelles in bacteria include thylakoids, the photosynthetic membranes of cyanobacteria [1], and magnetosomes of magnetotactic bacteria [2]. Overexpression of viral membrane proteins can also result in the formation of cytoplasmic membrane structures in bacteria [3, 4]. Furthermore, production of intracellular membrane vesicles in *Escherichia coli* can be triggered by expression of *Acholeplasma laidlawii* lipid glycosyltransferases [5]. Outside of these intriguing examples, intracellular membranes are rare in the majority of bacterial cells, making them attractive systems for cell-based vesicle production.

The only bacteriophages known to have a lipid envelope around their protein capsids are the members of the *Cystoviridae* family [6]. Pseudomonas phage phi6 infects Gram-negative plant-pathogenic *Pseudomonas* species [7, 8] and is the type member of this family [9]. Phi6 has three double-stranded RNA genome segments (S, M, and L) inside its triple-layered virion [10, 11]. Around the innermost core is a nucleocapsid surface shell composed of protein P8 [12–14]. The lipid-protein envelope around the nucleocapsid [6] consists of phospholipids derived from the host cytoplasmic membrane (CM) [15] and the following five viral membrane proteins: the major envelope protein P9, fusogenic protein P6, spike protein P3, putative holin protein P10, and minor membrane protein P13 [13, 16–18].

Phi6 has a lytic lifecycle [8, 19] and the envelope is acquired inside the host cytosol [20]. Several hypotheses have been presented for the mechanism of phi6 envelopment [21, 22] but the exact pathway is still unknown. Early studies on nonsense mutants of phage phi6 suggested that the major envelope protein P9 and the non-structural protein P12 are the only proteins needed for phi6 virion envelopment [23]. P12 and P9 are expressed consecutively from the S segment [24], and this genomic organization is highly conserved among known cystoviruses [25]. P9 has a molecular weight of 9.5 kDa and a putative transmembrane region at amino acids 51–66 [24]. In natural phi6 infection, P9 is likely to be delivered and attached into the CM via its transmembrane region. Recently, P9 was used as a fusion partner for eukaryotic membrane proteins to enhance their expression in an *E. coli* membrane [26].

How P12 facilitates viral envelopment is not known. However, several roles have been proposed, including assisting the other phi6 membrane proteins to the correct pathway [27], stabilizing membrane proteins, acting as a protease inhibitor [21], and a role as a lipid transporter [22]. Co-expression of phi6 proteins P9 and P12 in *Pseudomonas syringae* leads to the formation of low-density P9 particles [21]. Sarin et al. [28] demonstrated that phi6-specific vesicles are also formed in *E. coli* bacteria expressing P8, P9, and P12. Lately, this *E. coli* system was used to produce synthetic lipid-containing scaffolds and to co-localize P9-tagged enzymes or marker proteins to such intracellular structures [29].

The aim of this study was to identify the minimum protein components needed for phi6-specific vesicle formation in bacteria and the specific requirements for the localization of P9-tagged heterologous proteins into the vesicles. We demonstrate that phi6 P9 can trigger the formation of low-density membrane vesicles in *E. coli* in the absence of any other viral protein and that P9 is selectively incorporated into such vesicles. In addition, our data shows that P9 fusion proteins are directed into these membrane structures. However, the stable production of the fusion proteins requires P12, which protects against proteolytic action. A bacterial vesicle production system based on phi6 provides the possibility to produce lipid vesicles containing heterologous proteins for different biotechnological and biomedical applications.

## Materials and methods

### Bacterial strains and plasmids

*Escherichia coli* JM109 [30] was used as a cloning strain and *E. coli* BL21(DE3) [31] as an expression strain. Cells were grown at 37 °C at 200 rpm shaking in L-broth [1% (w/v) Bacto tryptone, 0.5% (w/v) Bacto yeast, and 0.5% (w/v) sodium chloride] unless otherwise stated. 150 µg/ml ampicillin, 25 µg/ml kanamycin and/or 50 µg/ml streptomycin were added to the cultivation media when bacteria contained pRSETa, pET28a + and/or pCDF-1b based plasmids, respectively (Table 1).

**Table 1:** Plasmids

Plasmid	Vector*	Proteins encoded	Resistance	Reference
pLM659	pT7T3-19U	P8, P12, P9, and P5	Ampicillin	[32]
pDS4	pCDF-1b	P9	Streptomycin	This study
pOL2	pET28a+	P12	Kanamycin	This study
pOL5	pCDF-1b	P12 and P9	Streptomycin	This study
P9-GFP	pRSETa	P9-GFP fusion	Ampicillin	[26]

* pT7T3-19U (Pharmacia); pET28a + (Novagen); pCDF-1b (Novagen); pRSETa (Invitrogen)

Pseudomonas phage phi6 S segment *genes 12* and *9* were amplified using pLM659 [32] as a template in a polymerase chain reaction (PCR) (Tables 1 and 2). For protein expression, the amplification products were ligated to pET28a + or pCDF-1b vectors (Table 1). Genes encoding proteins P12 and P9 were cloned separately and consecutively under the T7 promoter. The resulting plasmids (pOL2, pDS4, and pOL5) (Table 1) were used for the expression of P9 and P12 in cis and in trans. Plasmid P9-GFP [26] is a derivative of the pRSETa vector and encodes P9 with a C-terminally fused green fluorescent protein (GFP) (Table 1).

**Table 2:** Primers

Primer	Sequence	Tm, °C*	Plasmid
P9_5′_*Nco*I	5′-TACAGTCCATGGCTATGCCATTTCCTCTGGTAAAG-3′	62.2	pDS4
P9_3′_*Hin*dIII	5′-TAGCACAAGCTTAGGCCAGAAAAGGGATG-3′	62.0	pDS4
P12_5′_*Nco*I	5′-ATACCATGGTTATCGGTCTCCTGAAG-3′	62.3	pOL2, pOL5
P12_3′_*Eco*RI	5′-TTAGAATTCATTACGGAACATCCTTACG-3′	60.0	pOL2
P9_3′_*Hin*dIII_R	5′-GCGCAAGCTTAGGCCAGAAAAGGGATGTTG-3′	66.2	pOL5

* Melting temperatures (Tm) were calculated using the ThermoFisherScientific Tm calculator, which is based on the modified Breslauer’s thermodynamics method [33]

### Expression and purification of lipid-protein vesicles

Expression and isolation of lipid-protein vesicles were based on protocols introduced by Sarin et al. [28] and Laurinavičius et al. [34]. *E. coli* BL21(DE3) cells were grown to an optical density at 550 nm (OD_550_) of 0.6–0.8 and expression of phi6-specific proteins was induced with 1 mM isopropyl β-d-1-thiogalactopyranoside (IPTG) for 20 h at room temperature (RT). Cells collected by centrifugation were suspended in 20 mM Tris–HCl pH 7.5, 150 mM NaCl and disrupted with a French press at 800 pounds per square inch (psi), corresponding to approximately 5.5 MPa. After centrifugation (Sorvall F-28/50 rotor, 8200*g*, 20 min, 4 °C), the vesicles were precipitated from the supernatant with 9% (w/v) polyethylene glycol (PEG) 6000 and 5% (w/v) NaCl for 1 h at 4 °C under magnetic stirring and collected by centrifugation as above. The resulting pellet was washed gently with milli-Q water and dissolved overnight in 10 mM KH_2_PO_4_, 1 mM MgCl_2_ on ice. Any insoluble material was removed by centrifugation (Hitachi Koki himac CT15RE centrifuge, T15A61 rotor, 9600*g*, 10 min, 4 °C) prior to the following analyses. The PEG precipitation procedure was omitted when samples containing P9-GFP fusion protein were analyzed.

For flotation centrifugation, approximately 3 ml of the PEG-precipitated sample or the cleared lysate in approximately 77% (w/v) sucrose was loaded on a 0.5-ml cushion of 80% (w/v) sucrose in 20 mM Tris–HCl pH 8.5, 150 mM NaCl, 10 mM MgCl_2_ buffer. The sample was covered with 2-ml layers of 57%, 48%, 39%, and 30% (w/v) sucrose solutions in the same buffer. Following flotation centrifugation (Sorvall TH641 rotor, 210,000*g*, 22 h, 20 °C unless otherwise stated), 1-ml fractions were collected with a piston gradient fractionator (BioComp) and the pellet was resuspended in 1 ml of 68% (w/v) sucrose, 20 mM Tris–HCl pH 8.5, 150 mM NaCl, 10 mM MgCl_2_ buffer. An additional rate-zonal centrifugation step (Sorvall TH641 rotor, 125,000*g* for 1 h 50 min at 20 °C) was performed before flotation centrifugation when higher vesicle purity was desired.

### Microscopy methods

*Escherichia coli* BL21(DE3) thin sections were prepared as follows: cells with selected plasmids (pDS4, pOL2, pCDF-1b; Table 1) were grown to OD_550_ 0.6–0.7 and expression of phi6-specific membrane proteins was induced as described in “Expression and purification of lipid-protein vesicles”. Collected cells were suspended in L-broth and fixed with 2.5% (v/v) glutaraldehyde, 20 mM KPO_4_ pH 7.2 in L-broth in foil at RT for 1 h. Cells were washed gently three times with 20 mM KPO_4_ pH 7.2 in L-broth and the final glutaraldehyde-fixed cell pellet was stored with supernatant overnight on ice. The fixed cells were osmium-tetroxide treated, dehydrated, plastic embedded in Epon, thin-sectioned, and post-stained with uranyl-acetate by the Electron Microscopy Unit of the University of Helsinki. The P9 vesicle fraction from the flotation centrifugation analysis was concentrated 10× using Amicon Ultra-4, Ultracel 30 K concentrator (Merck Millipore Ltd., UFC803024), the concentrated sample, in 20 mM Tris–HCl pH 7.5, 150 mM NaCl, was applied on a carbon coated, glow discharged EM grid for 1 min, and stained with uranyl-acetate for 30 s. The thin sections and P9 vesicles were analyzed using transmission electron microscopy (TEM; Jeol JEM-1400, 80,000 V). For the fluorescent microscopy *E. coli* BL21(DE3)(P9-GFP)(pOL5) cells were grown and protein expression induced as described in “Expression and purification of lipid-protein vesicles”. Suspension of the cell pellet in 20 mM Tris–HCl pH 7.5, 150 mM NaCl was visualized using Olympus BX50 microscope with FITC (fluorescein isothiocyanate) filter and visible light.

### Analytical methods

Densities of the 1-ml fractions from the flotation centrifugation analyses were determined from three independent experiments by measuring the weight of three 100 µl subfractions from each fraction. The fractions were precipitated with trichloroacetic acid (TCA) and analyzed by sodium dodecyl sulfate polyacrylamide gel electrophoresis (SDS-PAGE). The SDS-PAGE gels were stained with Coomassie, Sudan black, or both and imaged using a ChemiDoc Touch imager (BioRad) or used for Western blotting. Polyclonal antibodies (pAb) against P12 (1:13,333; GeneCust) and phi6 (1:2500) and a monoclonal antibody against GFP (1:25,000; ab291, Abcam) were used as primary antibodies. HRP-labeled anti-rabbit pAb (1:125,000; Sigma Aldrich) or anti-mouse pAb (1:5000; PI-2000; Vector Laboratories Inc.) were used as secondary antibodies. Chemiluminescence was detected using a Western Lightning ECL Kit (Perkin-Elmer) and ChemiDoc Touch imager (BioRad) with 600 s of exposure time. Mass spectrometric analyses of P12 were performed in the Proteomics Unit of the University of Helsinki using liquid-chromatography–mass spectrometry × 2 (LC–MS/MS). Quantitative analyses of the EM thin sections was done using Aida Image Analyzer v 4.5.

## Results

### P9 induces vesicle formation in the absence of P12

#### Detection of spherical structures in *E. coli* thin sections

**Fig. 1:**
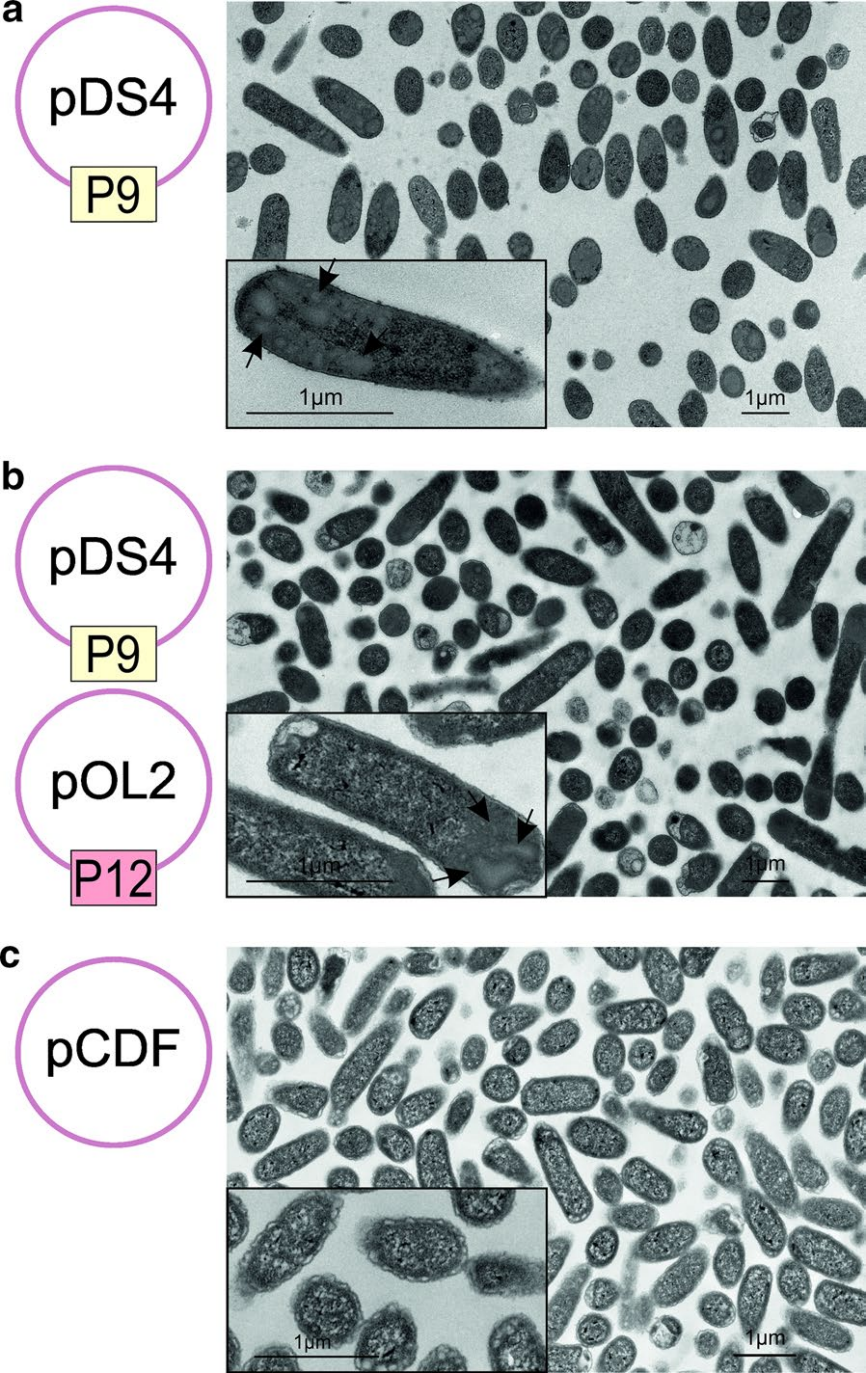
Thin sections of *E. coli* cells expressing P9 and P12. *E. coli* BL21(DE3) cells expressing phi6 proteins P9 and P12 were fixed with glutaraldehyde, embedded in Epon, thin sectioned, and analyzed by transmission electron microscopy (TEM). Representative TEM images of cells expressing the major envelope protein P9 from plasmid pDS4 (**a**), P9 and the non-structural protein P12 from plasmids pDS4 and pOL2, respectively (**b**), and control cells containing vector plasmid pCDF-1b (**c**). Each scale bar represents 1 µm. The arrows in **a** and **b** point to structures specifically identified in cells expressing P9

The phi6 major envelope protein P9 and the non-structural protein P12 are the minimum requirements for viral envelope formation in the *Pseudomonas* host [23]. To further investigate P9- and P12-driven vesicle formation [21, 28], we expressed P9 alone (pDS4) and together with P12 (pDS4 and pOL2; Table 1) in *E. coli* BL21(DE3). Thin-sectioned BL21(DE3) cells were analyzed by TEM (Fig. 1). Expression of P9 alone (Fig. 1a) or P9 together with P12 (Fig. 1b) resulted in formation of spherical structures not detected in the BL21(DE3)(pCDF-1b) control cells (Fig. 1c). These large, evenly stained, spherical areas appeared to be polarized and more abundant in cells expressing P9 only than in the cells producing both P9 and P12 (Fig. 1a, b; detected in 66 and 37 cells out of hundred cells, respectively). The areas of these structures varied from 0.01–0.72 µm^2^ (Fig. 1a, b). However, these numbers are likely underestimates as the putative P9-specific structures were not evenly distributed in the cells and the display of the structures strongly depends on the position of slicing. Also, it cannot be distinguished if an identified area is composed of multiple densely packed structures or present a single structure.

#### P9 vesicle detection by flotation centrifugation

**Fig. 2:**
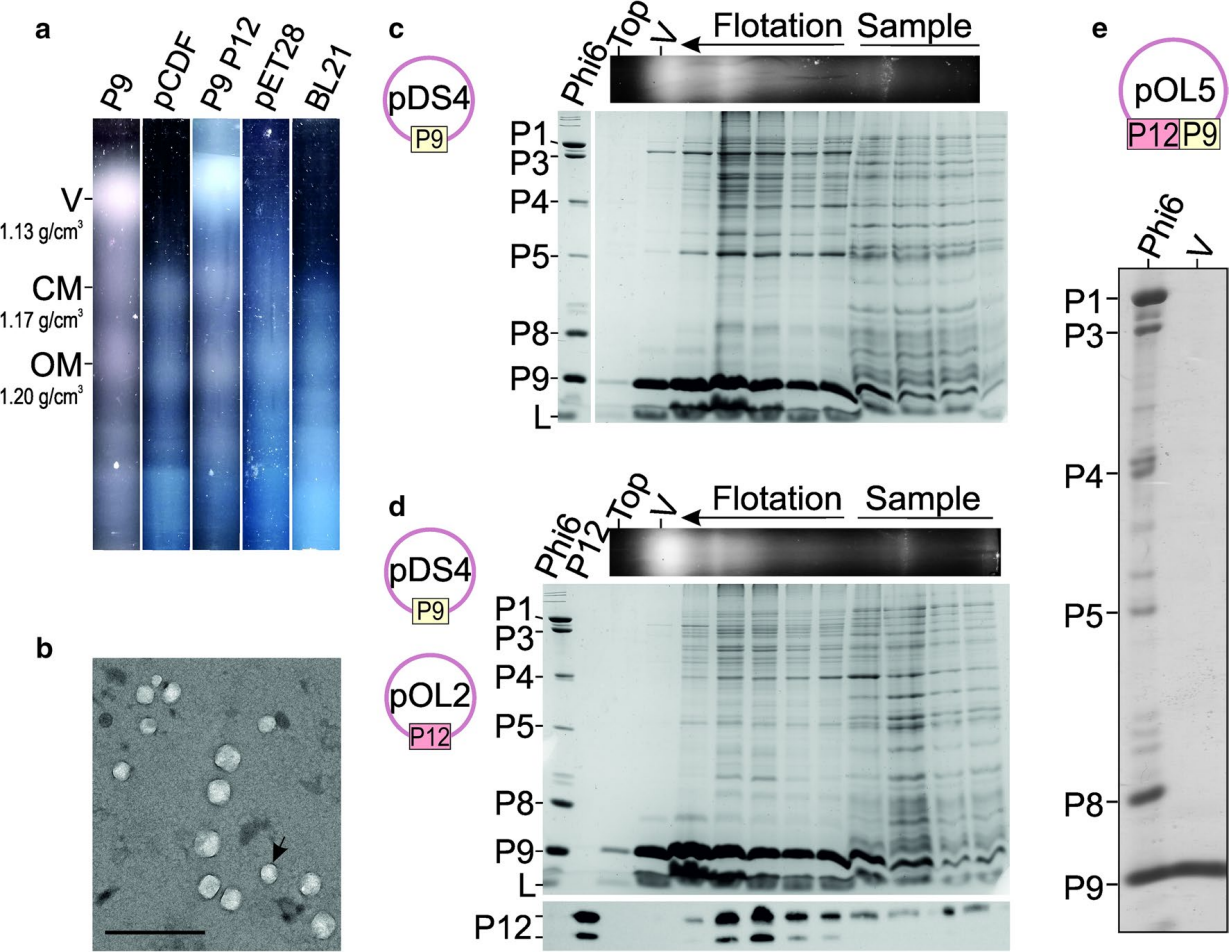
Membrane fractions of cells expressing phi6 proteins P9 and P12. The phi6 major envelope protein P9 and non-structural protein P12 were co-expressed in *E. coli* overnight. Solid sucrose was added to PEG-precipitated cell lysates to obtain approximately 77% (w/v) sugar concentration, and the samples were analyzed by equilibrium flotation centrifugation (**a**–**d**). After centrifugation, light scattering was detected under visible light (**a**). The positions of the OM, CM, and P9 vesicle (V) fractions and their densities are indicated on the left, and the proteins expressed or the corresponding vector controls are indicated above the tube images (**a**). **b** Negatively stained P9 vesicles produced in *E. coli* BL21(DE3)(pDS4) analysed using TEM. The scale bar corresponds to 500 nm. **c**, **d** Analysis of the flotation gradient fractions (top panel) by SDS-PAGE. The plasmids used and proteins produced are presented on the left. The gels were stained with Coomassie and Sudan Black to identify proteins and lipids (L), respectively (**c**, bottom panel; **d**, middle panel) or by Western blotting using P12-specific antibodies (**d**, bottom panel). The position of the P9 vesicle fraction is indicated (V; top panels; **c**, **d**). Phi6 represents purified phi6 virions (**c**, **d**) and P12 purified recombinant P12 (**d**). The main structural proteins of phi6 (**c**, **d**) and the two mobility forms of the non-structural protein P12 (**d**) are indicated on the left. **e** P9 vesicles (V) were purified from PEG-precipitated *E. coli* BL21(DE3)(pOL5) lysates using rate-zonal and equilibrium flotation centrifugation at 4 °C. The plasmid used is presented above, and the main structural proteins of the phi6 standard on the left (**e**)

Electron microscope analyses suggested that expression of P9 induces formation of intracellular membrane structures (Fig. 1). To clarify the nature of these structures, we analyzed the membrane fractions of the corresponding *E. coli* cells using flotation centrifugation analysis. Lysed Gram-negative bacteria typically produce two light scattering zones in equilibrium centrifugation, namely an upper zone representing the CM and the lower zone representing the outer membrane (OM) [5, 35, 36]. Corresponding light scattering zones were detected when lysates of cells expressing P9 or control cells harboring the pCDF-1b vector were analyzed (Fig. 2a). Interestingly, the expression of P9 resulted in the formation of an additional membrane fraction, detected as a light scattering zone above the CM fraction (Fig. 2a). The density of this fraction was approximately 1.13 g/cm^3^ in sucrose, corresponding to a lipid-to-protein ratio of approximately 1:1 (w/w; assuming a pure membrane protein density of 1.28 g/cm^3^ and a lipid density of 1.02 g/cm^3^ [37]). The densities of the CM and OM fractions were approximately 1.17 g/cm^3^ and 1.20 g/cm^3^, respectively. We analyzed the light scattering, low-density fraction using TEM and detected spherical, vesicular structures approximately 100 nm in diameter (Fig. 2b). Only P9 and lipids were detected in these low-density upper fractions (Fig. 2c). These data confirms the presence of P9-specific lipid vesicles. P9 was also abundant in the CM fraction, supporting the idea that P9 is initially produced and delivered to the CM. However, the CM and OM fractions contained a plethora of other cell-derived background proteins, whereas the low-density fraction was composed almost merely of P9 and lipids (Fig. 2c).

#### P12 does not associate with P9 vesicles

Flotation analysis of the lysates of cells expressing P9 and P12 resulted in a similar pattern of light scattering zones as observed for the lysates of P9-expressing cells (Fig. 2a). Low-density light scattering material composed of P9 and lipids was detected irrespective of the expression system and the presence of P12 (Fig. 2c, d). Western blot analysis of the gels using anti-P12 antibody revealed two closely migrating P12-specific bands (Fig. 2d), both of which were confirmed to represent P12 by LC–MS/MS analysis. P12 was not associated with the P9 vesicle fractions but could be detected in the CM fractions (Fig. 2d). This suggests that P12 has affinity for lipids or proteins associated with the CM, but is not directly associated with P9 vesicles that are predicted intermediates in the phi6 envelope assembly pathway. However, this result does not exclude the possibility that P12 interacts with P9 at the CM.

#### Purification of P9 vesicles

To further analyze the composition of the low-density P9 vesicles, we purified vesicles from lysates of *E. coli* BL21(DE3)(pOL5) cells expressing P12 and P9 *in cis*, using both rate-zonal and flotation centrifugation (Fig. 2e). No cellular proteins could be detected in the uppermost fractions of purified P9 vesicles, indicating that formation of P9 vesicles is a highly selective process (Fig. 2e).

### Introduction of heterologous P9-GFP fusion protein

#### P9-GFP is susceptible to protease degradation

**Fig. 3:**
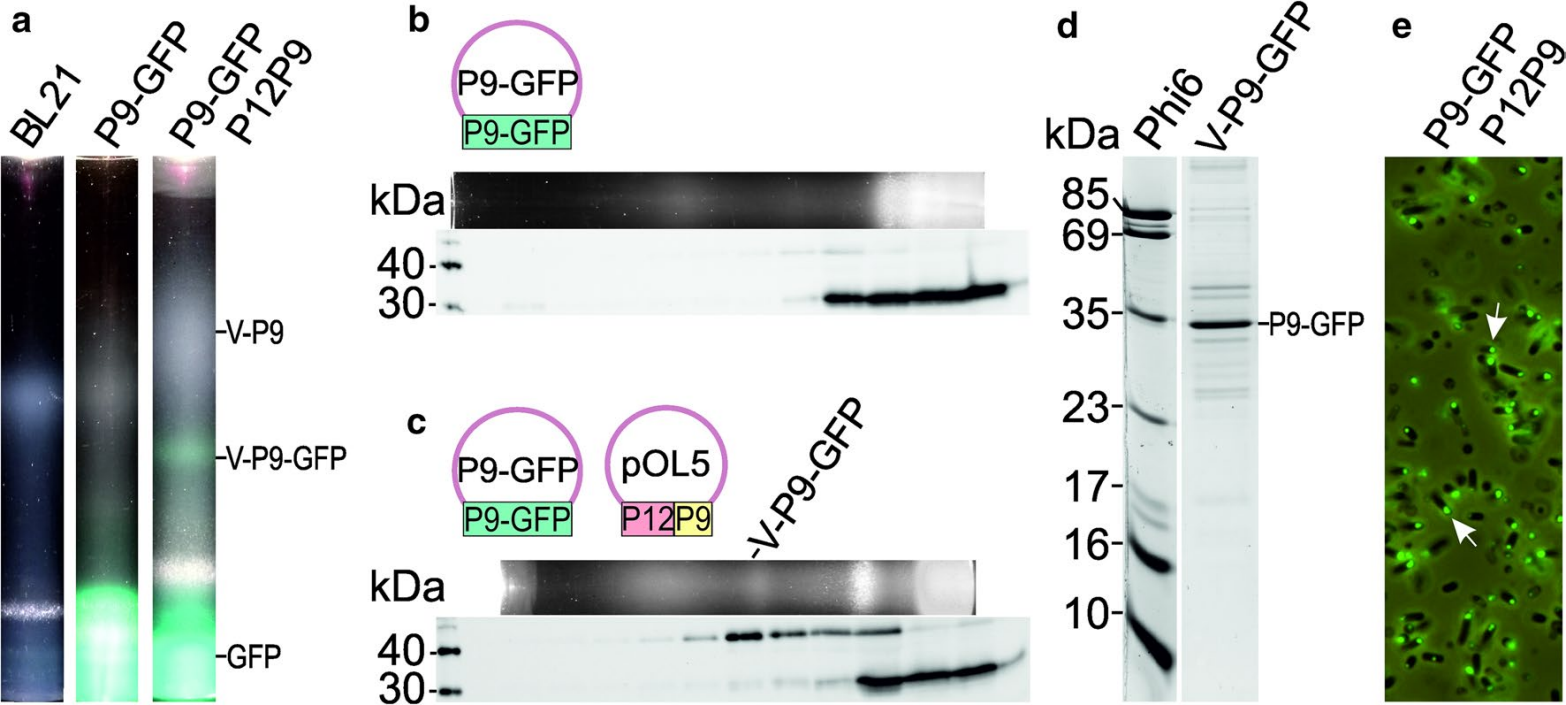
Expression of P9-GFP fusion protein. P9-GFP fusion protein was expressed alone or together with wild type P9 and the non-structural protein P12 in *E. coli* BL21(DE3) cells. Solid sucrose was added to the lysate (**a**–**c**) to obtain approximately 77% (w/v) sugar concentration for the equilibrium flotation centrifugation analysis. After flotation centrifugation, light scattering was detected under visible light (**a**). The positions of the P9 and P9-GFP vesicle fractions (V-P9 and V-P9-GFP, respectively), and the position of soluble GFP are indicated on the right. The proteins expressed are indicated above the tube images. BL21 indicates *E. coli* BL21(DE3) control cells (no phi6 protein expression). **b**, **c** Analysis of the flotation gradient fractions (top panel) by Western blotting using an anti-GFP antibody (bottom panel). The expressed proteins and the plasmids used are presented above. The position of the P9-GFP vesicle (V-P9-GFP) is indicated (**c**, top panel). The 40- and 30-kDa bands from MagicMark™ XP Western Protein standard (ThermoFisher Scientific) are indicated on the left (**b** and **c**; bottom panels). **d** Coomassie-stained SDS-PAGE gel of P9-GFP vesicles purified from *E. coli* BL21(DE3)(P9-GFP)(pOL5) cells by rate-zonal and equilibrium flotation centrifugation (42 h). Phi6 represents purified phi6 virions. The molecular weights of selected phi6 structural proteins are indicated on the left (**d**). **e** *E. coli* BL21(DE3)(P9-GFP)(pOL5) cells were visualized using Olympus BX50 microscope and FITC (fluorescein isothiocyanate) filter and visible light. The proteins expressed are indicated on the top, the arrows indicate fluorescent foci inside the cells

Previously, the phi6 major envelope protein P9 was used as an expression partner to enhance heterologous membrane protein production in *E. coli* [26]. In this system, GFP was used as an indicator for proper folding of the expressed membrane proteins [26, 38]. We analyzed the effect of the GFP-tag on formation of P9-specific vesicles. Lysates of *E. coli* BL21(DE3) cells expressing the P9-GFP fusion protein were analyzed by flotation centrifugation analysis (Fig. 3a, b). The observed pattern of the light scattering zone was similar to that observed for the lysate of *E. coli* BL21(DE3) control cells, except for an intense green zone that appeared at the bottom of the tube (Fig. 3a). This suggested that the expressed GFP was not associated with lipids (Fig. 3a). Western blot analysis using anti-GFP antibody revealed that the bottom fractions contained a GFP-specific polypeptide of approximately 30 kDa (Fig. 3b). This likely represents the 26.9 kDa GFP-tag, which is proteolytically cleaved from the 36.4 kDa P9-GFP fusion protein (Fig. 3b).

#### P12 facilitates P9-GFP vesicle formation

To test the hypothesis that P12 could act as a protease inhibitor [21], we co-expressed P9-GFP with P12 and P9 (pOL5). This resulted in formation of a green light scattering zone in the flotation centrifugation analysis (Fig. 3a). The density of the collected green fraction was slightly higher than the density of the P9 vesicle (1.17 g/cm^3^ versus 1.13 g/cm^3^) and corresponded to the density of the CM of Gram-negative bacteria (*E. coli*, 1.15–1.17 g/cm^3^ [35]; *P. syringae*, 1.18 g/cm^3^ ± 0.02 [15]). The full-sized P9-GFP fusion protein (36.4 kDa) could be identified in these fractions by Western blot analysis using anti-GFP antibody (Fig. 3c). Although both P9 and P9-GFP were expressed in the cell, only the P9-GFP fusion protein was detected in the fractions (Fig. 3d). The expression of P12 and P9 separately with P9-GFP confirmed that the formation of the P9-GFP vesicle was dependent on P12 (Additional file 1).

#### Purification of P9-GFP vesicles

To determine whether these vesicles are specific to P9-GFP and thus distinct from random CM vesicles, the P9-GFP vesicles produced in *E. coli* BL21(DE3)(P9-GFP)(pOL5) were expressed and visualized by fluorescent microscope and purified using rate-zonal and flotation centrifugation. The purified P9-GFP vesicle fraction contained predominantly the 36.4 kDa P9-GFP fusion protein (Fig. 3d) and *E. coli* cells expressing these P9-GFP vesicles contained fluorescent foci polarized to the ends of the cells (Fig. 3e).

## Discussion

### Phi6 P9 can induce vesicle formation in *E. coli*

In this study, we showed that the phage phi6 major envelope protein P9 induces production of intracellular membrane structures in *E. coli* in the absence of any other viral protein. Expression of P9 in *E. coli* resulted in the production of spherical cytoplasmic structures (Fig. 1). When the membranes of these cells were isolated it was possible to identify a low-density fraction distinct from the bacterial CM and OM (Fig. 2). This fraction contained vesicles composed of P9 and lipids (Fig. 2b, c); no other proteins were detected (Fig. 2e). Thus, the vesicles produced in this system appeared to be more uniform in composition than those obtained previously in comparable *E. coli* expression systems [5, 28]. The detected membranous structures inside *E. coli* cells (Fig. 1) and the high P9 specificity of the vesicles (Fig. 2e) indicate that the P9 vesicles are not simply byproducts of the cell lysis procedure but separate membrane structures selectively produced within the cell due to the activity of P9.

### P9-GFP vesicle production is enhanced by P12

GFP was originally isolated from *Aequorea* jellyfish [39] and has been used in a wide range of biotechnological applications, including detection of recombinant protein expression, localization of proteins within cells, or as an indicator for the proper folding of membrane proteins [26, 29, 38]. We used a P9-GFP fusion protein to determine if it is possible to incorporate heterologous proteins into the P9-specific vesicles. Previously, Myhrvold et al. [29] detected fluorescent foci inside cells expressing a P9-YFP (yellow fluorescent protein) fusion protein and P12. However, it was not clear whether these foci were part of the CM or if there were cellular membrane proteins associated with P9-specific membrane patches. In our study, we could not isolate P9-GFP vesicles when P9-GFP was expressed in *E. coli* in the absence of other viral proteins (Fig. 3a). One possible explanation for this could be the relatively large size of GFP (26.9 kDa) compared to P9 (9.5 kDa), which could result in steric hindrance preventing potential interactions between neighboring P9 subunits and thus the formation of the vesicles. However, co-expression of P9 and P9-GFP did not result in the formation of P9-GFP vesicles (Additional file 1) and no P9 was associated with the P9-GFP vesicles produced by co-expressed P9, P12, and P9-GFP (Fig. 3d). Another possible explanation could be the instability of the fusion protein, as GFP fusion proteins are prone to proteolysis [26, 40]. In support of this hypothesis, we detected a GFP-specific polypeptide of approximately 30 kDa in the bottom fractions of the flotation centrifugation tubes. Interestingly, the addition of the non-structural protein P12 in the expression system rescued the full-length P9-GFP fusion protein (Fig. 3 and Additional file 1). Co-expression of P9-GFP, P9, and P12 resulted in the formation of a floating GFP-containing fraction (Fig. 3a, c). The density of this fraction was similar to that of the CM fraction of Gram-negative bacteria [15, 35]. To exclude the possibility that P9-GFP was part of the normal CM, we analyzed the cells in fluorescent microscopy (Fig. 3e) and purified the P9-GFP vesicles using rate-zonal and equilibrium centrifugation (Fig. 3d). The cells had inner fluorescent foci and the P9-GFP was clearly enriched in the purified vesicles, suggesting that the P9-GFP vesicles were formed selectively and were not byproducts of mechanical CM disruption.

## Conclusions

In this study we produced bacteriophage phi6-specific lipid-protein vesicles in *E. coli* BL21(DE3) cells using the phi6 major envelope protein P9 and the non-structural protein P12. Expression of P9, a small viral protein with a transmembrane region, triggered intracellular membrane formation in bacterial cells that do not normally have internal membrane structures and thus likely do not possess mechanisms to support the production of such features. P9 could also direct a heterologous protein fused to P9 into membrane structures. However, the production of intact fusion protein was dependent on the co-expression of the non-structural protein P12, known to be involved in viral envelope biogenesis. In our *E. coli* expression system, P12 most likely acts as an inhibitor of proteolytic action or as a molecular chaperon that protects the full-sized P9-fusion protein. Thus, P12 may have potential use as a facilitator of recombinant membrane protein production, although additional research is needed on this topic. The P9-based vesicle production system described here is an excellent tool to study the molecular mechanisms of biological vesicle formation. We envision that the system will likely have future use in biotechnology and biomedicine in applications such as drug or heterologous protein targeting and delivery.

## Additional file

**Additional file 1.** Expression of P9-GFP fusion protein with P9 and P12. P9-GFP fusion protein was expressed together with either wild type P9 (**a**) or the non-structural protein P12 (**b**) in *E. coli* BL21(DE3) cells. Cells were collected and disrupted and solid sucrose was added to the cleared lysate to obtain approximately 77% (w/v) sugar concentration for the equilibrium flotation centrifugation analysis. The contents of the flotation centrifugation tubes were fractionated and the fractions were TCA precipitated and analyzed by Western blotting using an anti-GFP antibody. The expressed proteins and the plasmids used are presented above. The 40- and 30-kDa bands from MagicMark™ XP Western Protein standard (ThermoFisher Scientific) are indicated on the left (bottom panel).

## Abbreviations

CM: cytoplasmic membrane
GFP: green fluorescent protein
kDa: kilo dalton, 1 dalton = atomic mass unit
LC–MS/MS: liquid-chromatography–mass spectrometry/mass spectrometry
OD_550_: optical density at 550 nm wavelength
OM: outer membrane
pAb: polyclonal antibody
PEG: polyethylene glycol
rpm: revolutions per minute
RT: room temperature
SDS-PAGE: sodium dodecyl sulfate polyacrylamide gel electrophoresis
TEM: transmission electron microscopy
TCA: trichloroacetic acid
