# Supplementary figures and images for: Microbial production of lipid-protein vesicles using enveloped bacteriophage phi6
Source: Microb Cell Fact. 2019 Feb 7;18:29. doi: 10.1186/s12934-019-1079-z (PMC6366064; doi:10.1186/s12934-019-1079-z)

## Additional file 1

A

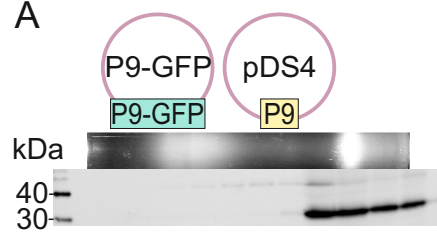

B

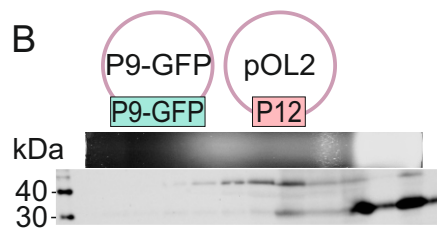

Supplement: Supplementary file 1 — Additional file 1. Expression of P9-GFP fusion protein with P9 and P12. P9-GFP fusion protein was expressed together with either wild type P9 (a) or the non-structural protein P12 (b) in E. coli BL21(DE3) cells. Cells were collected and disrupted and solid sucrose was added to the cleared lysate to obtain approximately 77% (w/v) sugar concentration for the equilibrium flotation centrifugation analysis. The contents of the flotation centrifugation tubes were fractionated and the fractions were TCA precipitated and analyzed by Western blotting using an anti-GFP antibody. The expressed proteins and the plasmids used are presented above. The 40- and 30-kDa bands from MagicMark™ XP Western Protein standard (ThermoFisher Scientific) are indicated on the left (bottom panel). [file 12934_2019_1079_MOESM1_ESM.pdf]
